# Medical Education and the London University

**Published:** 1907-12-14

**Authors:** T. Gregory Foster

**Affiliations:** Provost.


### Editor's Letter-Box.

### MEDICAL EDUCATION AND THE LONDON UNIVERSITY.

University College, Gower Street, London, W.C.
December 4, 1907.

*To the Editor of* The Hospital.

Dear Sir,—I beg to acknowledge with thanks the receipt of your issue of Saturday, November 30. I have read with interest the article on "Medical Education and the London University."

While I sympathise with the view expressed in the article that it is much to be regretted that the scheme for the establishment of an Institute of Medical Sciences has proved abortive, I feel obliged to call attention to three points likely to give rise to misunderstanding.

The article states "the original scheme in the minds of its creators was that of a single institute in a central part of the Metropolis, accessible to every medical school and favouring none, it being a necessary corollary to the erection of such a centre that as long as University and King's Colleges were directly or indirectly connected with any individual hospital they also should abandon the teaching of the preliminary and intermediate medical studies." In reply to this statement I desire to point out that a scheme involving the surrender of the teaching of the preliminary and intermediate medical studies at University and King's Colleges has never been proposed, and consequently never been sanctioned.

The incorporation of University College in the University took place simultaneously with the creation of a separate corporation for University College Hospital and the School of Advanced Medical Studies connected therewith. It has been arranged that a like severance of the hospital and Medical School at King's College shall take place when King's College is incorporated.

The incorporation of University College in the University took place on January 1 last, and on that day the connection between the hospital and the Medical School on one hand and the College on the other was severed. There were certain transitional arrangements made by the Commissioners which permitted the Medical School to use part of the College buildings until their own new buildings were ready. The separation is a complete one, and has been loyally carried out, both in spirit and letter. Under these crcumstances I do not understand the statement in your article which runs as follows: "How University College was incorporated without really severing its intimate connection with the hospital on the other side of Gower Street."

The third statement to which I desire to call attention is made towards the end of the article. It is to the effect that, previous to the election of Professor Starling to the Senate, University College or Hospital were "already over-represented with six members." Such a statement is misleading, and is not in accordance with strict facts.

Under the statutes of the University, University College and King's College have each two direct representatives on the Senate. These are the only members of University College or King's College on the Senate who have any right to act as representatives of the institution. There were, previous to the election of Professor Starling, three professors connected with University College—namely, Professors Gardner, M. J. M. Hill, and Sir William Ramsay, who were members of the Senate; but they were members of the Senate as representatives of their respective faculties, sent by those faculties to act on their behalf and generally help in the governing of the University. I presume that the sixth member referred to in your number is Sir Thomas Barlow, who was elected by the graduates in medicine to represent them. Sir Thomas Barlow is no longer on the teaching staff of University College, because the subjects of medicine and surgery are no longer taught at University College, but at University College Hospital Medical School. In any case, however, Sir Thomas is a representative of the "graduates," and not of his school.

I venture to hope that you will publish this letter, thus correcting the misapprehensions likely to arise from the form in which the statements are made in your article.

I am, yours truly,
T. Gregory Foster, *Provost.*

.·. It is quite correct, as Dr. Gregory Foster states, that a scheme involving the surrendering of the teaching of the preliminary and intermediate studies at University and King's Colleges has never been proposed, and consequently never been sanctioned. That does not affect the statement as to the original scheme in the minds of its creators, and it is just the difference between the ideal, and what has occurred as a matter of actual fact, that has determined the change of opinion at the larger medical schools with reference to the whole question of concentration. It is also true that in the letter University College and University College Hospital are separate institutions; but the fact remains that they are too close to each other ever to be separate. University College can never be a neutral centre, as the hospital on the other side of the road will always be the most obvious place at which the students who have done their preliminary and intermediate medical studies at University College will take up their advanced courses for the final examinations. In addition, in the Act of Incorporation of University College, in the second Schedule, Part I., paragraph 5, it is stated that the persons constituting the Governors of the hospital may include, among others, "such of the Fellows, Governors, or Life Governors of University College, London, as may within six months after the appointed day signify in writing addressed to the Secretary of the Hospital their desire to be members of the Corporation of the Hospital." Further, it is, we believe, a matter of common knowledge that a scheme is on foot for the purchase of a new athletic ground for the clubs of University College and University College Hospital Medical School, for which an appeal is being made by a committee consisting of representatives of both institutions.

This is sufficient evidence that the relations between University College and University College Hospital are of the most intimate character.

With reference to the representation of University College on the Senate, Dr. Gregory Foster only confirms the statement made in The Hospital. It cannot be maintained that only those members of University College who are elected under the statutes of the University can be considered as representing University College. There were when we wrote six members of the staff of University College or Hospital on the Senate, and the election of Professor Starling has made seven. This, in our opinion, is an excessive number.—[Ed. The Hospital.]

THE HOSPITAL December 14, 1907.

